# Experiencing the impact of weight loss on work capacity prior to initiation of a weight loss program enhances success

**DOI:** 10.1186/1550-2783-8-S1-P2

**Published:** 2011-11-07

**Authors:** Mike Greenwood, Michelle Mardock, Brittanie Lockard, Jonathan Oliver, Sunday Simbo, Andrew Jagim, Julie Kresta, Claire Baetge, Peter Jung, Majid Koozehchian, Deepesh Khanna, Chris Rasmussen, Richard Kreider

**Affiliations:** 1 Exercise & Sport Nutrition Lab. Texas A&M University, College Station, TX 77843, USA

## Background

A number of psychological interventions have been employed prior to and/or during exercise and weight loss interventions in an attempt to influence exercise adherence, compliance, and/or success. However, few studies have evaluated whether these types of efforts influence program efficacy. The purpose of this study was to determine whether having sedentary and overweight individuals experience the impact of losing weight on work capacity prior to initiation of an exercise and/or weight loss program would influence weight loss success.

## Methods

Fifty-one sedentary women (35±8 yrs, 163±7 cm; 90±14 kg; 47±7% body fat, 34±5 kg/m^2^) were randomized to walk on an AlterG Anti-Gravity Treadmill^®^ (AG) at 3 mph at 100% and 80% of body mass or were entered into a weight loss program directly (WL). Participants were then randomized to participate in the Curves(C) exercise and weight loss program or the Weight Watchers (W) weight loss program for 16-wks in order to examine whether this strategy may be more effective depending on the type of weight loss program employed. Participants in the C program were instructed to follow a 1,200 kcal/d diet for 1-week, 1,500 kcal/d diet for 3 weeks, and 2,000 kcals/d diet for 2-weeks consisting of 30% carbohydrate, 45% protein, and 30% fat. Subjects then repeated this diet. Subjects also participated in the Curves circuit style resistance training program 3 days/week and were encouraged to walk at brisk pace for 30-min on non-training days. This program involved performing 30-60 seconds of bi-directional hydraulic-based resistance-exercise on 13 machines interspersed with 30-60 seconds of low-impact callisthenic or Zumba dance exercise. Participants in the W group followed the W point-based diet program, received weekly counseling at a local W facility, and were encouraged to increase physical activity. Four-day dietary records, the International Physical Activity Questionnaire (IPAQ), and dual energy X-ray absorptiometer (DEXA) determined body compositionmeasurements were obtained at 0, 4, 10, & 16 weeks and analyzed by MANOVA with repeated measures. Data are presented as changes from baseline for the WL and AG groups, respectively, after 4, 10, and 16 weeks.

## Results

No significant differences were observed in energy intake or macronutrient intake among those in the AG or WL groups. The amount of vigorous PA performed at each data point in the AG group was significantly greater throughout the study (WL 953±1,221, 844±653, 1,338±1,767, 1,266±1,535; AG 803±1,282, 1,332±1,719, 1,286±1,974, 1,579±2,091 MET-min/wk, p=0.01) despite even distribution of participants among supervised and non-supervised exercise programs. Overall, MANOVA revealed a significant time by intervention effect (p=0.02) in body composition variables. Univariate analysis revealed that both groups lost a similar amount of weight (WL -2.8±2.1, -5.3±3.4, -5.9±4.4; AG -2.3±1.1, -4.3±2.4, -5.1±3.5 kg, p=0.40) and fat mass loss (WL -2.0±6.1, -2.4±6.4, -4.1±7.8; AG -2.1±5.7, -4.4±5.7, -5.2±6.4 kg, p=0.43) while changes in fat free mass (WL -0.3±5.4, -2.1±5.2, -1.5±5.2; AG -0.3±5.1, 0.3±4.7, 0.2±4.6 kg, p=0.08) and percent body fat (WL -1.0±5.9, -0.2±6.1, -1.7±6.6; AG-1.5±6.9, -3.9±7.5, -4.5±7.6 %, p=0.07) tended to be more favorable in the AG group.

## Conclusion

Results indicate that experiencing the impact of losing weight on work capacity prior to initiation of an exercise and/or weight loss program has a positive impact on increasing vigorous activity and changes in body composition. More research is needed to examine whether use of this strategy more often during a weight loss program may affect adherence and/or efficacy of different types of weight loss programs.

## Funding

Supported by Curves International (Waco, TX) and AlterG, Inc. (Fremont, CA)

